# Plasma proteomics analysis of vitamin C supplementation in people with type 2 diabetes

**DOI:** 10.1038/s41387-026-00447-5

**Published:** 2026-06-27

**Authors:** T. Van Grunsven, GD Wadley, SA Mason

**Affiliations:** 1 School of Exercise and Nutrition Sciences, Deakin University, Melbourne, VIC Australia; 2 Institute for Physical Activity and Nutrition, School of Exercise and Nutrition Sciences, Deakin University, Geelong, VIC Australia

**Keywords:** Type 2 diabetes, Type 2 diabetes

## Abstract

**Background:**

Evidence from randomised controlled trials shows that vitamin C (VC) supplementation may improve cardiometabolic health outcomes in people with type 2 diabetes (T2D). Plasma proteomics may help to further enhance our understanding of VC’s therapeutic effects and biological mechanisms in T2D. Therefore, our aim was to explore the effects of VC supplementation on the plasma proteome in people with T2D.

**Methods:**

A double-blind, placebo-controlled, crossover trial was undertaken in people with T2D, who were administered 1000 mg/day VC or placebo for 4 months. Plasma proteins in 26 participants (22 male, 4 female, age 62.6 ± 6.5 years, HbA1c 60 ± 11 mmol/mol [7.6 ± 0.7%]) were quantified by liquid chromatography/mass spectrometry with data-independent acquisition. Differential protein expression was assessed for VC post-supplementation vs. control [pooling of all non-active conditions].

**Results:**

Twelve proteins were significantly downregulated and three were upregulated following VC supplementation (|log2-fold change [FC]| ≥ 0.5; false discovery rate<0.05). These included proteins with functions in innate immunity, carbohydrate digestion, unfolded protein binding, and muscle contraction. Protein candidates that had the greatest magnitude decrease (|log2-FC| > 1) with VC supplementation were alpha-amylase 1 (AMY1), heat shock protein 75 kDa (TRAP1), myosin-1 (MYH1), and serum amyloid protein A1 (SAA1). Pathway enrichment revealed underrepresentation of unfolded protein binding, ATP binding, attenuation phase, and the innate immune system following VC supplementation. TRAP1, alpha-actin-1, Heat shock 70 kDa protein-1, and CD166 antigen were associated with previously reported improved blood-pressure outcomes following VC supplementation.

**Conclusions:**

VC supplementation significantly altered abundance of numerous proteins in plasma, with functions relating to the innate immune system, dietary carbohydrate digestion, and protein folding chaperone activity. We identified novel proteins responsive to VC supplementation and protein-clinical improvement correlations in people with T2D that require further exploration to understand their biological significance.

## Introduction

Diabetes mellitus affects ~10.5% of adults aged 20–79 years globally, the majority (~90–95%) with type 2 diabetes (T2D) [1]. Prevalence has risen steadily over the past 25 years and is projected to continue increasing [1]. T2D is associated with macro- and microvascular complications that contribute to disability, premature mortality, and economic burden [2].

T2D is typified by progressively defective β-cell insulin secretion, insulin resistance, and dysregulated hepatic glucose production [2, 3]. Hyperglycaemia and mitochondrial dysfunction promote excessive reactive oxygen species production leading to oxidative stress [4], which impairs glucose metabolism and accelerates the development of complications [4, 5]. Vitamin C (VC), a potent water-soluble antioxidant, neutralises reactive oxygen species, regenerates other antioxidants, and supports nitric oxide production, improving vascular function and insulin sensitivity [2, 6, 7]. VC supplementation may therefore mitigate oxidative stress-related dysfunction in T2D, warranting investigation of its effects on the plasma proteome.

Evidence from clinical trials has shown that VC may improve glycaemic control, blood pressure, lipid profiles, and oxidative stress biomarkers in T2D [8–10], although individual responses vary [2]. This variability highlights the need for biomarkers to guide personalised treatments, support early diagnosis, and predict complications [3, 11, 12]. Rapidly responsive biomarkers could also shorten assessment of treatment efficacy, reducing prolonged hyperglycaemia and β-cell decline [3].

Multi-omics approaches offer powerful tools to assess variation between patients [11, 13]. Proteomics in particular, reflects gene expression influenced by lifestyle and provides functional insight into disease drivers [11, 12, 14]. Plasma-based proteomics is accessible and reflects systemic metabolism [12]. Some plasma proteins are already utilised in mainstream pathology tests, such as C-reactive protein (CRP) for assessment of coronary disease risk [15]. Advancements in high-throughput technologies have enabled large-scale studies of low-abundance proteins [16]. For example, proteomic profiling of 1,221 participants identified 146 plasma proteins predictive of future T2D, validating aminoaylase-1 as a biomarker up to 15 years before disease onset [16].

Two studies in people without diabetes found that VC supplementation altered plasma polypeptide patterns [17], and plasma protein concentrations of Ceruloplasmin, Alpha-1-anti-trypsin, Angiotensinogen, Vitamin D binding protein, Plasminogen, and the Complements C3 and C9 [18]. To our knowledge, no studies have investigated the impact of VC or other antioxidants on the plasma proteome in people with T2D.

Studies of pharmacological, lifestyle, and surgical interventions in adults with T2D consistently show changes in metabolically relevant proteins [12, 19–25]. For example, Apolipoprotein A-I, downregulated in T2D and linked to retinopathy [20], is upregulated by metformin, chiglitazar, exercise, or bariatric surgery [19, 20, 22, 25]. These findings demonstrate the utility of proteomics for identifying treatment-responsive biomarkers and elucidating disease mechanisms.

The aim of this study was to identify differentially expressed plasma proteins resulting from 4 months of VC supplementation in adults with T2D.

## Methods

### Study design

A 4-month double-blinded, placebo-controlled, crossover intervention was conducted in individuals with T2D (#ACTRN12616000276459) as previously described by Mason et al. [10]. Ethics approval was obtained from Deakin University Human Research Ethics Committee, project number 2015-319. All participants provided written informed consent prior to study commencement. All methods were performed in accordance with relevant institutional guidelines and regulations and in accordance with the Declaration of Helsinki. Participants in the treatment group took 500 mg ascorbic acid twice daily, while the placebo group took capsules of identical appearance. A washout period of minimum 1 month (mean 46 ± 16 days) separated the treatments. Treatment order was randomised and concealed by a third party with no direct involvement in the study [10].

Forty-three individuals underwent clinical screening to determine eligibility and 31 participants were enroled [10]. Inclusion criteria included T2D diagnosis, aged 35–75-years, management of T2D with diet or oral anti-hyperglycaemic medications, stable HbA1c between 48 and 86 mmol/mol (6.5–10%), total cholesterol ≤6.5 mmol/l, high-density lipoprotein cholesterol ≥0.9 mmol/l, triglycerides ≤4.0 mmol/l, body mass index <35 kg/m^2^, systolic blood pressure <160 mmHg, and diastolic blood pressure <90 mmHg [10]. Exclusion criteria included currently taking vitamin supplements, presence of heart murmur, bleeding disorder, haemochromatosis, comorbid cardiovascular disease, renal or liver disease, smoking, pregnancy or planning a pregnancy. Our sample size was suitably powered (>90%) for the primary outcome measure of postprandial incremental area under the curve [10], however, given the exploratory nature of the current study, no strict sample size was further established. At each time point, blood samples were collected for proteomic analysis using venipuncture into lithium heparin containing tubes after an overnight fast (10–12 h), and the plasma was separated via centrifugation (15 min, 3000 × *g*) and stored at −80 °C until analysis. Data collection methods for other clinical and biochemical measurements have been reported previously [10].

### Sample preparation

Liquid-chromatography-mass-spectrometry (LCMS)-grade chemicals were used for all steps. Plasma samples (10 µl) were diluted with a lysis buffer comprising 1% sodium dodecyl sulphate and 100 mM 4-(2-hydroxyethyl)-1-piperazineethanesulfonic acid to obtain a protein amount of ~10 µg. Samples were then prepared using single-pot, solid-phase-enhanced sample preparation (SP3) with Sera-Mag^TM^ carboxylate-modified magnetic SpeedBeads (Cytvia, Marlborough, MA, USA) at a ratio of 20:1 beads:protein (wt/wt) [26]. For digestion of proteins into peptides, trypsin was added to samples at 50:1 protein:trypsin mass ratio and incubated at 37 °C 1200 rpm in a ThermoMixer (Eppendorf SE, Hamburn, Germany) for 18 h. Samples were then cooled at room temperature and placed on magnetic racks. Peptide desalting and clean-up utilised stop and go extraction (STAGE) tips prepared in pipette tips with 2 layers of Empore^*TM*^ (3 M, St. Paul, MN, USA) styrenedivinylbenzene-reverse-phase-sulphonate membrane. Samples were dried via vacuum centrifugation and then resuspended in 20 µl of 2% acetonitrile with 0.1% trifluoracetic acid (TFA).

### Liquid chromatography and dual-mass spectrometry (LC-MS/MS) analysis

Samples were analysed using an UltiMate 3000 RSLCnano system interfaced with an Orbitrap Exploris™ 240 Mass Spectrometer (ThermoFisher Scientific, Waltham, MA, USA). Peptide samples (2 µl) were loaded onto a C18 nanoViper trap column for 3 min at a flow rate of 15 µl/min using 2% acetonitrile and 0.1% TFA, then separated on a C18 nanoViper analytical column at a flow rate of 250 nl/min. Mobile phases included 0.1% formic acid (buffer A) and 80% acetonitrile with 0.1% formic acid (buffer B). Gradient conditions were as follows: 0–2 min 2.5% B, 2–2.1-min 8% B, 2.1–122-min 32% B, 122–125-min 45% B, 125–133-min 99% B, and 133–150-min 2.5% B. A ThermoFisher NanoSpray Flex^TM^ Ion source was employed with a static spray voltage. The positive ion voltage was set at 1900 V, and negative ion voltage set to 1500 V. The ion transfer tube was maintained at 275 °C.

Data independent acquisition (DIA) was used to acquire data during a 150-min scan using Thermo Fisher XCalibur software. Full scan (MS1) Orbitrap resolution was set to 60,000, with a scan range of 350–1200 m/z. DIA (MS2) acquisition parameters were as follows: precursor mass range between 400 and 1000 m/z; isolation window 12 m/z and 0 m/z overlap; window placement optimisation enabled; normalised collision energy 30%; Orbitrap resolution 30,000; RF lens 70%; custom AGC target 3000%. Maximum injection time was set to automatic. Data were acquired in centroid mode with positive polarity.

### Data analysis

The resulting spectra were processed using Spectronaut® v.19.0 (Biognosys AG, Schlieren, Switzerland) to identify and quantify peptides and proteins. A Library-free DirectDIA^TM^ analysis was undertaken, incorporating FASTA files for homo sapiens (UP000005640_9606) and universal contaminants [27]. Pulsar search parameters included trypsin/P cleavage with a maximum of 2 missed cleavages; a 7–52 peptide length search range; carbamidomethyl (cysteine) as a fixed modification, and acetyl (N-terminal) and methionine oxidation as variable modifications. An FDR of 0.01 was selected as a threshold for PSMs, peptides, and proteins. The directDIA+ (Deep) workflow was run on the data set; and DNN predicted ion mobility was set to “Auto”. DIA analysis identification required a precursor q-value cut-off of 0.01 with a posterior error probability of 0.2. An experiment-wide protein *q*-value cutoff was set to 0.01 and run-wise protein q-value cutoff set at 0.05. Single hits, defined by stripped sequence, were not excluded.

To maximise our exploratory investigation, we filtered out precursors with >40% missing data across samples and used the Background Signal imputation in Spectronaut [28]. The background signal imputation strategy involves imputing by using the underlying MS signal for the detected peak. We also undertook the same analysis with no imputation or filtering using the Identified (*Q* value) in Spectronaut to investigate possible imputation effects and to consolidate our candidate list. The MaxLFQ algorithm [29] was used for label-free quantification. Cross-run normalisation utilised a retention time-dependent local regression model as described by Callister et al. [30].

Differential regulation analysis involved two-sided paired t-tests and combined both MS1 and MS2 data. Protein candidates were identified when their absolute log2 fold change (FC) between conditions was ≥0.5 and false discovery rate (FDR) < 0.05 in the filtered/imputed analysis [31]. Tier 1 protein candidates also displayed directionally consistent statistical significance (FDR < 0.05) in the unfiltered/non-imputed analysis and were |log2-FC| < 0.5 different between analyses. Tier 2 protein candidates were directionally similar and |log2-FC| < 0.5 different across analyses but did not achieve statistical significance in the unfiltered analysis (FDR > 0.05). Proteins only significant in the main analysis but displaying directional difference and/or with |log2-FC| > 0.5 difference compared to the unfiltered analysis were discarded as candidates, due to uncertainty of effects of imputation driving their findings.

Although data were collected at four time points (pre- and post- supplementation for both VC and placebo), we focused on pooled contrasts for VC post-supplementation vs. VC pre-supplementation, placebo pre-supplementation, and placebo post-supplementation conditions to investigate effects of VC against an averaged background of all non-active conditions (Vitamin C vs Control). We combined paired t-test *p*-value data generated from Spectronaut across these contrasts considering the correlated data [32] and then pooled their log2-FC using weighted average by inverse *p*-value. FDR correction was made using Benjamimi Hochberg correction. Average log2FC and −log10 FDR values were used to generate volcano plots in VolcaNoseR [33]. Local Network Cluster Enrichment, Gene Ontology (GO) enrichment, and Reactome analyses were conducted using STRING database [34].

We also investigated possible carry-over and period effects in our cross-over design via a sensitivity analysis limited to each cross-over period separately. We firstly compared all period-1 non-active time points to period-1 post-supplementation VC (followed by a comparison of period-2-only data) using unpaired t-tests in Spectronaut. Given that period-1 is devoid of carry-over effects, we used this analysis to further consolidate our candidates list. A second sensitivity analysis was conducted based on a per-protocol basis, with participants who varied their medication use (*n* = 5) or did not achieve compliance standards with VC and/or placebo supplements (*n* = 2) being removed for the analysis [10].

Correlations between log2 transformed abundances of the identified protein candidates and select clinical outcomes previously shown to significantly improve with VC supplementation (total postprandial glucose area under the curve [−10%], average 24 h glucose [−0.8 mmol/L], time spent in hyperglycaemia [−2.8 h per day], systolic blood pressure [SBP; −7 mmHg], and diastolic blood pressure [DBP; −5 mmHg]) [10] were assessed using repeated measured correlations with STATA/SE18.0, which accounts for within-individual associations across repeated measures [35]. Both unadjusted and partial correlations adjusted for time-variant covariates, including body weight, per cent body fat, estimated glomerular filtration rate, accelerometer-determined physical activity energy expenditure, cross-over period, and medication compliance were determined [10]. Of note, we also reported three additional postprandial glycaemic outcomes that significantly improved in our previous study [10]. However, we only chose the abovementioned glycaemic measures for comparison, as they capture the key orthogonal aspects of glycaemic control (overall, postprandial, and time-based exposure), without considerable redundancy.

Lastly, we undertook a post-hoc analysis to explore potential proteins at baseline (pre-supplementation) as possible predictors of intervention responses. For this analysis, we undertook Spearman’s correlations between baseline protein abundances and the intervention response ([VC post supplementation minus VC pre-supplementation] minus [placebo post-supplementation minus placebo pre-supplementation]) for the abovementioned significantly changed clinical outcomes. We undertook enrichment analyses using String database to further probe any patterns of enriched GO ontology terms that might suggest baseline protein clusters that are predictive of intervention effects in the absence of individual protein-specific relationships. Correlations were subsequently undertaken between top-enriched GO terms and intervention responses. For these correlations, approximate data normality and choice of either Spearman’s or Pearson’s correlations were determined based on visual inspection of Q-Q plots. Covariate adjustments were made for partial correlations, adjusting for age, sex, per cent body fat, baseline outcome values, and baseline vitamin C concentration. Other covariates added to the model that did not alter statistical findings were medication use and diabetes duration (data not shown).

## Results

### Baseline participant characteristics

The baseline characteristics of participants are shown in Table 1. Of the initial 31 participants, enroled, four withdrew due to family or work obligations or due to illness [10]. As a result of sample preparation complications causing substantial sample loss, data from one participant were excluded from the current analysis, resulting in a final sample of 26 participants for paired analyses.

**Table 1:** Baseline participant characteristics (*n* = 26).

Characteristic/measure	Mean ± standard deviation
Sex (male/female)	22/4
Age (years)	62.8 ± 6.5
Height (cm)	174.3 ± 9.6
Weight (kg)	87.6 ± 11.6
BMI (kg/m^2^)	28.8 ± 2.9
Body fat (%)	34.2 ± 5.2
Systolic blood pressure (mm Hg)	140.2 ± 10.4
Diastolic blood pressure (mm Hg)	85.7 ± 9.2
Duration of diabetes (years)	5.8 ± 4.8
Diabetes management (*n*):	
• Metformin	18
• Janumet (sitagliptin + metformin)	4
• Sulfonylurea	6
• SGLT2 inhibitor	1
• Lifestyle only	4
HbA1c mmol/mol (%)	60 ± 11 (7.6 ± 0.7)
Fasting glucose (mmol/l)	9.1 ± 1.7
Total cholesterol (mmol/l)	4.9 ± 1.4
LDL-C (mmol/l)	2.6 ± 1.1
HDL-C (mmol/l)	1.3 ± 0.3
Triglycerides (mmol/l)	2.1 ± 0.9
Plasma ascorbic acid (µmol/l)	40.3 ± 16.7
Estimated glomerular filtration rate (ml/min/1.73 m²)	79.0 ± 11.2
Albumin (g/l)	40.0 ± 2.2
Alanine aminotransferase (U/l)	31.5 ± 13.5

Data adapted from Mason et al. [10].

*SGLT2* sodium-glucose co-transporter 2, *HbA1c* haemoglobin A1c, *LDL-C* low density lipoprotein cholesterol, *HDL-C* high density lipoprotein cholesterol.

### Proteomics analysis

A total of 820 protein groups were detected across samples, with a median of 621 protein groups per sample. Data completeness was 75.3% across samples, with 78% of identified protein groups found in 50% of the samples (Supplementary Fig. S1). Original and normalised peptide signal responses are shown in Supplementary Fig. S2. Within-condition coefficients of variation for proteins and a Principal Component Analysis (PCA) plots of the different treatment conditions are shown in Supplementary Figs. S3 and S4, respectively. Data shows slightly higher percent coefficient of variation across participants during the VC Pre-supplementation condition (59%) vs. other conditions (range: 51–53%). There was considerable overlap between conditions in the PCA plot, with the first 3 PCA components explaining 30% of the variance.

Our main analysis on precursors filtered out with >40% missing data was undertaken on *n* = 555 protein groups after removal of likely contaminants, while the unfiltered/non-imputed analysis was conducted on *n* = 820 protein groups.

### Differential protein expression with VC supplementation

**Fig. 1:**
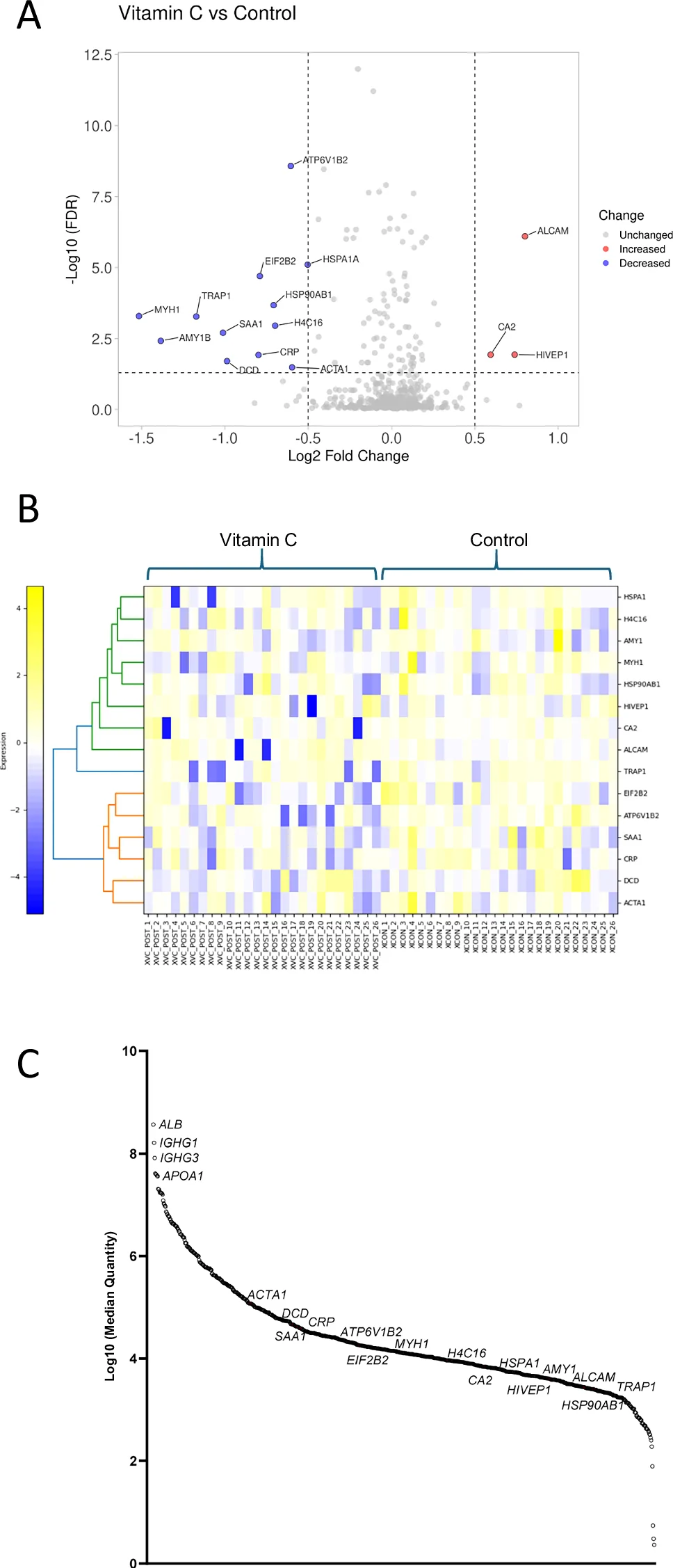
Protein candidates significantly regulated with vitamin C supplementation. Protein candidates regulated with vitamin C supplementation are shown in a volcano plot (**A**), a heat map (**B**), and in relation to the abundance of all identified proteins (**C**). In the volcano plot, proteins above the dashed horizontal line were significantly regulated (FDR < 0.05). Proteins to the right and left of the dashed vertical lines were regulated by |log2-FC| ≥ 0.5. Additional high abundance proteins, including albumin (ALB), immunoglobulin G (IGHG1, IGHG3) and apolipoprotein A1 (APOA1), are shown comparatively with the candidates in (**C**). The volcano plot was created using VolcaNoseR [33]; The heat map was created using Heatmapper [65].

One-hundred and one proteins were found to differ significantly (FDR < 0.05) between VC and Control in our main filtered/imputed analysis, however only 17 of these proteins were regulated by |log2-FC| ≥ 0.5 (Supplementary Table S1). Amongst these were 14 proteins that were downregulated and three proteins that were upregulated (Fig. 1A). Ten proteins were regarded as Tier 1 candidates, and five proteins were regarded as Tier 2 candidates (Table 2). Tier 1 proteins were found to be significant in both the main analysis as well as in the unfiltered/non-imputed analysis. Amongst these were nine that were decreased (serum amyloid protein A1 [SAA1], dermcidin [DCD], alpha-amylase 1B/C/A [AMY1B/C/A], myosin-1 [MYH1], translation initiation factor eIF2B subunit beta [EIF2B2], heat shock protein 75 kDa [TRAP1], heat shock protein HSP 90-beta [HSP90AB1], histone H4 [H4C16], and alpha actin 1 [ACTA1]), and one that was increased (CD166 antigen [ALCAM]). Tier 2 candidates found to be significant in the main analysis but not in the unfiltered/non-imputed analysis included three that were decreased with VC supplementation (C-reactive protein [CRP], V-type proton ATPase subunit B, brain isoform [ATP6V1B2], heat shock 70kda protein 1 [HSPA1A/B]) and two that were increased (carbonic anhydrase 2 [CA2] and zinc finger protein 40 [HIVEP1]). Two proteins, Lysine-specific demethylase 5B (KDM5B) and periostin (POSTN), were also found to be significantly decreased in our main analysis, however the effects observed in the main analysis were of a largely different magnitude and regulatory direction compared to the unfiltered/non-imputed analysis, and therefore these proteins were removed as candidates due to likely imputation-driven findings. Of note, our analysis was unable to distinguish the specific AMY1 and HSPA1 paralogs given an identical peptide sequence for each, and henceforward within the manuscript we refer to AMY1B/C/A as AMY1 and HSPA1A/B as HSPA1. Heat maps of protein candidates for VC vs. Control are shown in Fig. 1B. These candidate proteins were mostly located within the linear, mid-abundance range of the global proteome (Fig. 1C).

**Table 2:** Candidate plasma proteins altered following vitamin C supplementation compared with control.

Tier 1 candidates						
	Main analysis		Unfiltered/non-imputed analysis		Missing data^a^	Biological processes
Gene symbol	log2 fold change	FDR	log2 fold change	FDR		
MYH1	−1.516	5.055E-04	−1.445	1.894E-07	0%	Muscle contraction, sensory perception of sound
AMY1B/C/A	−1.385	3.778E-03	−1.431	8.358E-10	0%	Oligosaccharide metabolic process, carbohydrate metabolic process
TRAP1	−1.172	5.255E-04	−1.132	2.009E-03	30%	Negative regulation of cellular respiration, negative regulation of reactive oxygen species biosynthetic process, protein folding, translational attenuation
SAA1	−1.011	1.964E-03	−1.062	5.816E-03	0%	Positive regulation of cytokine production, acute-phase response, platelet activation, neutrophil chemotaxis, positive regulation of interleukin-1 production, positive regulation of cell adhesion, macrophage chemotaxis, lymphocyte chemotaxis, regulation of protein secretion, negative regulation of inflammatory response
DCD	−0.987	1.956E-02	−0.973	1.786E-02	0%	Antimicrobial humoral immune response mediated by antimicrobial peptide, defence response to bacterium, defence response to fungus, killing by host of symbiont cells, proteolysis
EIF2B2	−0.790	1.974E-05	−0.723	3.568E-04	8%	Cytoplasmic translational initiation, translational initiation, regulation of translational initiation, central nervous system development, response to heat, response to glucose, oligodendrocyte development, myelination, response to peptide hormone, T cell receptor signalling pathway
HSP90AB1	−0.707	2.092E-04	−0.318	1.687E-03	8%	Cellular response to heat, chaperone-mediated protein complex assembly, negative regulation of proteasomal ubiquitin-dependent protein catabolic process, positive regulation of protein localisation to cell surface, regulation of cell cycle, regulation of protein ubiquitination, response to unfolded protein
H4C16	−0.698	1.102E-03	−0.629	4.393E-04	0%	Chromatin organisation, negative regulation of megakaryocyte differentiation, nucleosome assembly,
ACTA1	−0.596	3.243E-02	−0.638	1.946E-03	0%	Mesenchyme migration, muscle contraction, positive regulation of gene expression, skeletal muscle fibre development, skeletal muscle thin filament assembly
ALCAM	0.801	7.838E-07	0.573	2.534E-02	36%	Adaptive immune response, axon extension involved in axon guidance, cell adhesion, heterophilic cell-cell adhesion via plasma membrane cell adhesion molecules, motor neuron axon guidance, signal transduction
**Tier 2 candidates**						
	**Main analysis**		**Unfiltered/non-imputed analysis**		**Missing data**^**a**^	**Biological processes**
**Gene symbol**	**log2 fold change**	**FDR**	**log2 fold change**	**FDR**		
CRP	−0.798	1.187E-02	−0.580	2.202E-01	0%	Vasoconstriction, acute-phase response, inflammatory response, negative regulation of macrophage derived foam cell differentiation, negative regulation of lipid storage, regulation of interleukin-8 production, positive regulation of superoxide anion generation, negative regulation of mononuclear cell proliferation, innate immune response
ATP6V1B2	−0.604	2.615E-09	−0.631	1.246E-01	37%	ATP metabolic process, proton transmembrane transport, regulation of macroautophagy, synaptic vesicle lumen and vacuolar acidification
HSPA1A/B	−0.503	7.806E-06	−0.197	4.308E-01	0%	ATP metabolic process, cellular response to heat, oxidative stress, and to unfolded protein, chaperone-mediated protein complex assembly, endoplasmic reticulum unfolded protein response, negative regulation of apoptotic process, cell growth, and protein ubiquitination, positive regulation of interleukin-8 production, NF-kappaB transcription factor activity, and tumour necrosis factor-mediated signalling pathway
CA2	0.594	1.164E-02	0.535	3.858E-01	5%	Angiotensin-activated signalling pathway, carbon dioxide transport, morphogenesis of an epithelium, positive regulation of cellular pH reduction, positive regulation of dipeptide transmembrane transport
HIVEP1	0.739	1.156E-02	0.434	5.863E-01	37%	BMP signalling pathway, regulation of transcription by RNA polymerase II

Tier 1 candidates were statistically significant in both analyses with directionally consistent effects. Tier 2 candidates were significant in the main analysis only but with directional consistency in the unfiltered/non-imputed analysis.

^a^Missing data is the percentage of data missing across all samples prior to imputation.

Our sensitivity analysis looking at each period separately largely confirmed our overall findings. All tier 1 candidates were statistically significant during the first period, which is devoid of any carryover effects (Supplementary Table S2). Many of these proteins were also significant in the second period and/or were regulated in a consistent direction in both periods. The small direction switches observed in some proteins (AMY1, DCD, HSPA1) are the expected consequence of analysing high‑variance proteomic data without pairing, not evidence of problematic period or carry‑over effects. Tier 2 candidates showed directional consistency during period 1 compared to findings of the main results, except for CRP. However, findings of the first period for CRP were confounded by a significant baseline difference in CRP between first period VC and placebo participants (VC vs. placebo baseline Log2-FC = 1.3, FDR < 0.05). Our “per protocol” sensitivity analysis, which removed participants who either changed their medications during the study or failed to comply with a 75% VC or placebo capsule compliance, also found very similar results to the main analysis (Supplementary Table S3). All tier 1 candidates remained statistically significant except for DCD, which was directionally consistent with the main findings. Three of the five tier 2 candidates also remained statistically significant, except for HIVEP1 and CA2, which were directionally consistent with the main analysis.

### Biological processes potentially affected by VC supplementation

**Fig. 2:**
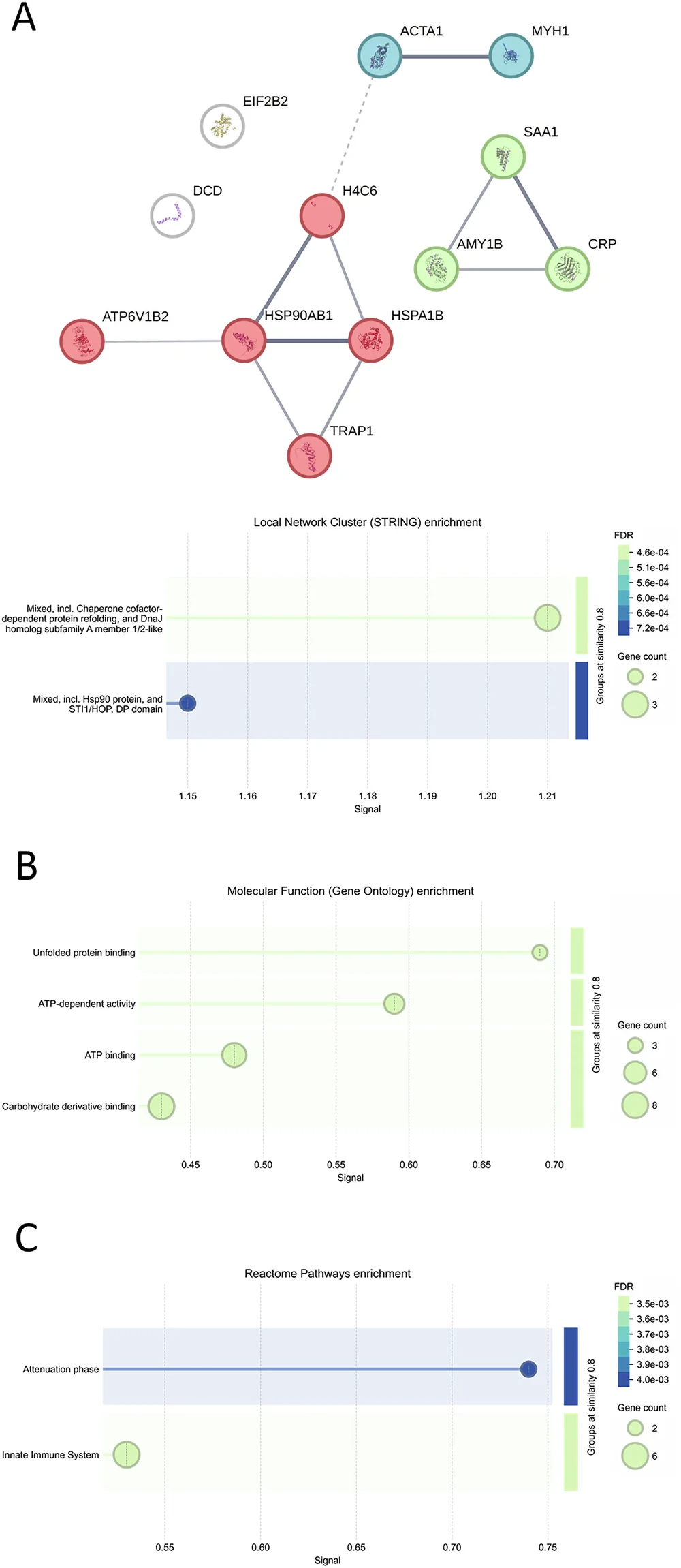
Underrepresented functional enrichments following vitamin C supplementation. Significantly underrepresented protein clusters found in plasma following vitamin C supplementation, along with significant STRING cluster enrichments (**A**). Significantly underrepresented Gene Ontology Molecular Function (**B**) and Reactome (**C**) found following vitamin C supplementation. *Strength* describes how large the enrichment effect is; *signal* is defined as a weighted harmonic mean between the observed/expected ratio and −log(FDR). FDR false discovery rate. Figures generated using String software [34].

To further explore the biological impact of proteins differentially expressed in response to VC supplementation, pathway enrichment analysis was conducted on all tier 1 and tier 2 candidates using the STRING database [34]. For these analyses, we collapsed AMY1 and HSPA1 isoforms down to a representative protein isoform so as not to inflate enrichments. When clustering candidate proteins using Markov algorithm clustering, three clusters were identified, characterised by: (a) Mixed, incl. Chaperone cofactor-dependent protein refolding, and DnaJ homologue subfamily A member 1/2-like; (b) acute-phase and (3) MYH1-ACTA1, which are muscle proteins (Fig. 2A). Underrepresentation of the GO Molecular Function pathways of unfolded protein binding and ATP-dependent activity were found (Fig. 2B); as were Reactome pathways of attenuation response and innate immune system (Fig. 2C).

### Protein correlations with clinical data

To further explore possible mechanistic associations and to probe for potentially useful biomarkers, we analysed correlations between plasma protein abundances of candidate proteins and significantly improved clinical outcomes. Table 3 summarises protein candidates that were significantly correlated with these improved clinical outcomes.

**Table 3:**
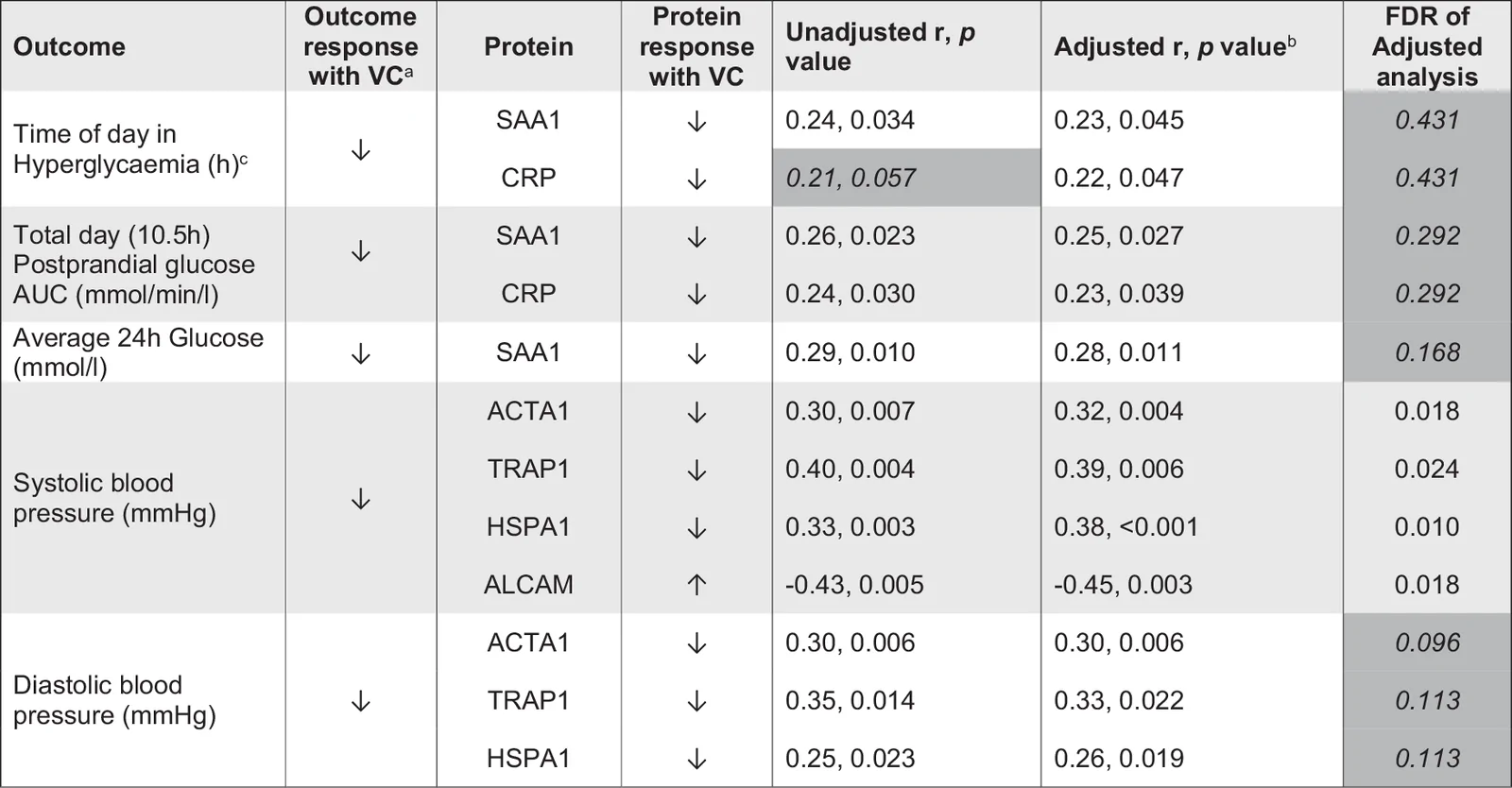
Protein candidates that correlated significantly with statistically significant clinical outcomes.

*FDR* false discovery rate.

^1^As reported in Mason et al. [10].

^2^Outcomes were adjusted for: body weight, per cent body fat, estimated glomerular filtration rate, accelerometer-determined physical activity energy expenditure, cross-over period, and medication compliance.

^3^log10 transformed. Darker shaded cells with italicised text represent non-significant data.

SAA1 and CRP correlated modestly but significantly with glycaemic-related outcomes both before and after adjustment for covariates, however these were no longer statistically significant after FDR correction. ACTA1, ALCAM, TRAP1 and HSPA1 correlated modestly with SBP both before and after adjustment for covariates, while ACTA1, TRAP1 and HSPA1 also correlated with DBP both before and after adjustment for covariates. After FDR adjustment, only the correlations between ACTA1, ALCAM, TRAP1 and HSPA1 and SBP remained significant.

**Fig. 3:**
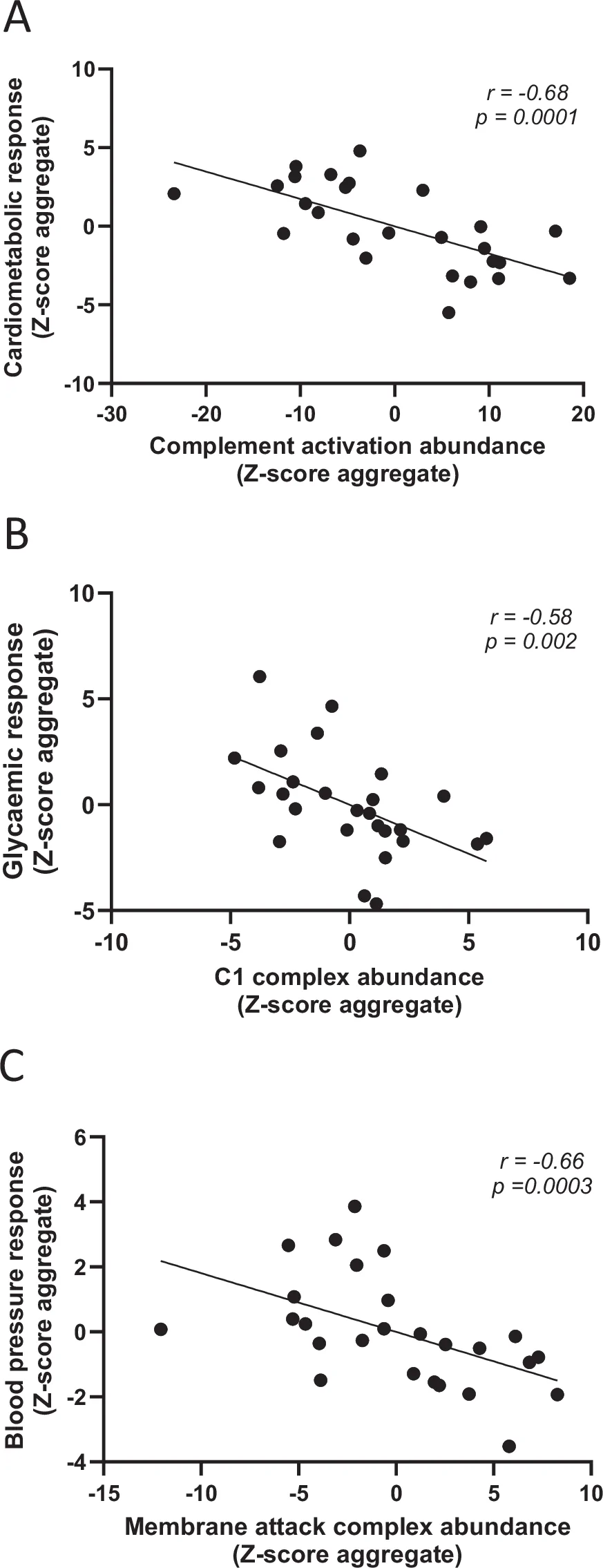
Association of baseline proteins and cardiometabolic outcomes with vitamin C supplementation. Partial correlation of aggregated protein abundance at baseline for enriched Gene Ontology term Complement activation (classical pathway; *n* = 30 proteins) and an aggregate of 3 x glycaemic- and 2 x blood pressure-related clinical outcomes representing a pooled ‘cardiometabolic response’ to vitamin C supplementation (**A**). Correlation of aggregate protein abundance at baseline for GO term C1 complex abundance (*n* = 5 proteins) and an aggregate of 3 x glycaemic-related clinical outcomes representing ‘glycaemic response’ to vitamin C supplementation (**B**). Correlation of aggregate protein abundance at baseline for GO term Membrane attack complex (*n* = 7 proteins) and an aggregate of 2 x blood pressure-related outcomes, representing ‘blood pressure response’ to vitamin C supplementation (**C**). Glycaemic measures include average 24 h glucose, total postprandial glucose area under the curve and time of day in hyperglycaemia; blood pressure outcomes include systolic blood pressure and diastolic blood pressure. Data shown has been adjusted for age, sex, per cent body fat, baseline aggregate of outcome measures and baseline vitamin C concentration.

When exploring potential baseline protein predictors of intervention responses, we did not find any specific proteins that were significantly correlated with the magnitude of intervention responses after FDR correction. However, we also looked at protein enrichments via String database. When looking at enrichments of the top 20 statistically correlated proteins for key outcome responses, significant enrichments correlating with improvements in both glycaemic-related and blood pressure outcomes with VC supplementation consisted of proteins involved in complement activation pathways and innate immune responses (Supplementary Table S4). When looking at cellular compartment enrichments, proteins in the complement C1 complex and membrane attack complex were particularly enriched for glycaemic-related and blood pressure outcomes, respectively. Given these findings, we further correlated a Z-score aggregate of all identified proteins with a known biological function in complement activation (classical pathway; GO:0006958; *n* = 30), complement C1 complex (GO:0005602; *n* = 5) and membrane attack complex (GO:0005579; *n* = 7) at baseline with intervention changes. We observed significant inverse correlations between proteins involved in complement activation (classical pathway) and all five clinical measures except time of day in hyperglycaemia (Supplementary Fig. S5). Given the similarity in outcome correlations, we further undertook a composite analysis correlation in which we generated a Z-score transformed composite of the 3 x glycaemic-related outcomes plus 2 x blood-pressure related outcomes (‘cardiometabolic response’ to VC supplementation) and correlated this with the protein aggregate for complement activation (classical pathway; GO:0006958). This analysis showed a significant inverse correlation between baseline levels of proteins involved in complement activation (classical pathway) and magnitude of improvement with VC supplementation before adjustment of covariates (*r* = −0.65, *p* < 0.001) and after adjustments (Fig. 3). Significant correlations were observed for complement C1 complex aggregate with changes in glycaemic outcomes and significant correlations were also found for membrane attack complex aggregate and changes in blood pressure outcomes with VC supplementation (Supplementary Fig. S6). Z-score composites for glycaemic-related outcomes and blood pressure-related outcomes were then separately correlated with baseline protein aggregate of C1 complex proteins and aggregate of membrane attack complex proteins, respectively, finding significant inverse correlations before covariate adjustments (*r* = −0.56, *p* = 0.003; *r* = −0.65, *p* < 0.001) and after covariate adjustments (Fig. 3).

## Discussion

The objective of this study was to identify differentially expressed plasma proteins resulting from 4-months VC supplementation in adults with T2D. A broad proteomics analysis from 26 participants in a placebo-controlled crossover trial revealed 15 proteins that were differentially expressed in response to VC. Numerous novel proteins not previously found to be altered with VC supplementation were identified. AMY1, SAA1, MYH1 and TRAP1 decreased by >2-fold following VC supplementation. While these relative fold changes are considered statistically strong, it is unclear what effect they might reflect clinically in people with T2D.

Reactome pathway analysis revealed decreased representation of the innate immune system following VC supplementation. This is notable because chronic overactivation of the immune system in T2D contributes to inflammation and tissue damage [36]. Several proteins with innate immune functions, including SAA1, CRP, ATP6V1B2, DCD, HSP90AB1 and HSPA1, significantly decreased following VC supplementation. SAA1and CRP are acute stress-response proteins that can activate immune cells and the production of pro-inflammatory cytokines [37]. CRP is an established risk factor for impaired glucose control and T2D [38, 39]. SAA1 is protective during acute immune responses, however sustained elevations in chronic inflammatory states, such as cardiovascular diseases, obesity and T2D, contribute to disease progression [40]. Thus, the attenuation of the acute-phase response proteins by VC may lower inflammatory burden and improve metabolic outcomes in people with T2D. Previous findings support our current findings, with randomised controlled trials reporting that VC supplementation can decrease serum CRP [41, 42]. Additionally, an intervention study using immunoassay found plasma SAA (reflecting SAA1 and SAA2) decreased by ~59% after VC supplementation [43]. SAA1 and CRP correlated modestly with previously reported improvements in glycaemic measures in our cohort, although after FDR correction they were no longer significant. [10]. Although these findings, along with biological plausibility, suggest that acute phase proteins such as SAA1 and CRP might be important therapeutic targets for VC supplementation in T2D, given the associative data, we cannot establish whether changes in these proteins lie on the causal pathway to glycaemic improvements or occur secondary to them.

HSPA1, TRAP1 and HSP90AB1 are stress-induced chaperones that were also found to decrease in plasma with VC supplementation. Enrichment analysis found depleted GO pathways of ATP-dependent protein folding chaperone and unfolded protein- and ATP-binding, and depleted Reactome pathways for attenuation phase. TRAP1, a mitochondrial heat shock protein (HSP), is known to increase in response to cellular stress and plays a role in redox homoeostasis [44]. HSPA1 (HSP70) is also upregulated in response to metabolic and cellular stress and inflammation, and increased levels of HSP70 were found to be associated with homoeostatic model assessment of insulin resistance (HOMA-IR) [45] and diabetes duration [46] in people with T2D. Elevated levels of HSP90AB1, a constitutive cytosolic HSP, have been implicated in impaired glucose metabolism and insulin signalling in skeletal muscle [47]. It is plausible that with VC supplementation, the oxidative and cell stress load becomes reduced, thus decreasing the activation of HSPs for mitigation of oxidative stress, misfolding of proteins and inflammation. This, therefore, results in a decreased need for chaperone upregulation and decreased extracellular release of HSPs such as HSPA1, TRAP1 and HSP90AB1. While this mechanism requires further experimental verification, the correlation of HSPA1 and TRAP1 with blood pressure outcomes in our cohort may lend further associative support to decreased cellular stress following VC supplementation. Indeed, HSP70 plasma concentration was shown to be significantly increased in patients with essential hypertension compared to controls [48]. Thus, improvements in cellular oxidative stress and inflammation with VC supplementation may further promote improvements in blood pressure [49], with associated decreases in HSPs.

Enrichment analysis via String also found a depletion in a cluster of proteins that are potentially of muscle origin. It is speculated that decreases in plasma levels of proteins, including MYH1 and ACTA1, reflect reduced muscle tissue damage, with their decreased cellular leakage. Given that people with T2D experience impaired muscle regeneration and skeletal muscle dysfunction [50], it is possible that improvements in inflammation and oxidative damage, such as in response to VC supplementation can modulate such effects. While there is currently limited research on effects of VC on skeletal muscle damage and protein leakage in people with T2D, some, but not all studies of VC supplementation have reported decreases in markers of skeletal muscle damage post intense exercise [51, 52]. Alpha actin increases in the blood following muscle injury [50] and may be elevated in people with T2D with complications [53]. Until further research undertakes targeted investigations on such mechanisms, our findings in relation to MYH1 and ACTA1 remain unclear, and our explanations remain speculative.

It is of interest to note that ACTA1 correlated with improvements in blood pressure. While this linkage is unclear, it might represent part of a broader signature of reduced cellular stress relating to decreases in cellular damage, oxidative stress, inflammation, and blood pressure, with decreased cellular leakage and/or release of proteins. Indeed, another protein found intracellularly—H4C16—a histone protein, was also found to be decreased in plasma with VC supplementation.

AMY1 was prominently downregulated following VC supplementation. This enzyme is primarily expressed in the salivary glands and is involved in the hydrolysis of glycosidic bonds in carbohydrate digestion [54]. Ascorbic acid has been shown to inhibit salivary amylase activity in vitro [55] and pancreatic amylase activity on raw and cooked starches [56, 57]. This inhibition could in theory promote a slowed rate of starch digestion and thus decreased postprandial hyperglycaemia. However, there is a lack of investigation to support this mechanism in vivo. Observational studies associating AMY1A copy number to insulin resistance and glucose homoeostasis have been mixed, with a review reporting a lack of strong evidence to support such a relationship [54]. Nonetheless, our findings offer a novel biomarker of change with VC supplementation, that might be of some relevance to glycaemic effects of VC supplementation in people with T2D.

Another protein found to decrease with VC supplementation was EIF2B2. EIF2B2 is a component of the translation initiation factor 2B complex, which can affect activation and/or repression of protein translation via GTP-binding. EIF2B activity has been found to be increased in pancreatic beta cells in response to glucose, leading to increased protein synthesis [58]. However, it is unclear how VC might impact on levels of this protein in plasma within the context of T2D.

The significance of the observed increase in ALCAM in plasma following VC supplementation is unclear. ALCAM is a cell adhesion molecule that has established roles in adaptive immunity and is involved in immune cell-endothelium interactions. A previous study reported increased serum and kidney concentrations of soluble ALCAM in patients with diabetes and diabetic nephropathy and was thus suggested as potential mediator of late complications of diabetic nephropathy [59]. Of note in our study, there was no change in blood kidney function markers, and in fact, ALCAM correlated inversely with the decrease in SBP observed. While the significance of VC effects on ALCAM in people with T2D remains to be further investigated, given the context of changes in other proteins and clinical effects we observed, it might reflect remodelling of immune-endothelial adhesion dynamics immune reprogramming rather than altered immune activation.

It is interesting that we did not detect significant changes in any major antioxidant enzymes in plasma in our study in response to VC supplementation. We detected catalase, SOD3, PRDX1, PRDX2 and GPX3, however, none were identified as candidates. Previous research involving VC supplementation in people with T2D has produced mixed outcomes with respect to effects on antioxidant enzyme levels in plasma [60]. However, most studies have explored enzyme activity levels rather than protein abundances, as it is the most functionally relevant outcome with respect to antioxidant enzymes.

While we found no specific proteins at baseline that were predictive of the magnitude of glycaemic or blood pressure response to VC supplementation, when examining the top‑ranked proteins showing nominal associations with treatment effects, pathway‑level enrichment analyses consistently highlighted complement activation (classical pathway) across our key cardiometabolic outcomes. Furthermore, enrichment of the C1 component complex was observed for glycaemic responses and membrane attack complex–related proteins for blood pressure responses, respectively. These findings suggest that individuals with T2D who have higher levels of these proteins might have greater magnitude improvements in glycaemic- and blood pressure-related outcomes with VC supplementation. While these findings should be interpreted cautiously, the consistency of these enrichments across outcomes suggests that baseline variation in innate immune pathways may contribute to inter‑individual heterogeneity in response to VC supplementation. These observations are hypothesis‑generating and warrant evaluation in larger, prospectively powered studies.

While some previous studies have utilised mass spectrometry-based proteomics to assess the relationship between proteins and serum VC concentration in humans [17, 18, 61], to our knowledge, this is the first study to investigate the effects of VC supplementation on the plasma proteome in people with T2D using LC-MS/MS proteomics. The robustness and reliability of our findings are supported by several key strengths of the study design and methodology. The initial intervention was a randomised, placebo-controlled, double-blind trial that reported significant improvements in some glycaemic- and blood-pressure-related outcomes with VC supplementation [10]. This enabled us to explore potential mechanistic associations of significantly altered proteins. Furthermore, we undertook complementary unfiltered/non-imputed and filtered/imputed analyses to look at effects of VC supplementation on the plasma proteome in people with T2D for purposes of broader biomarker discovery. High-abundance protein depletion was omitted to simplify the workflow and avoid potential bias, as depletion can remove bound low-abundance proteins, alter natural protein ratios and decrease consistency [15, 62].

Key limitations include a predominantly male cohort, limiting generalisability to females due to possible effects of sex hormones on protein expression [63]. The absence of a comparator group without diabetes prevents determining whether observed protein changes in response to VC are specific to T2D. Additionally, the cohort consisted of community-dwelling volunteers within a relatively narrow age range and with moderately controlled T2D. Protein responses may differ in a more heterogeneous population of people with T2D. Few participants had low baseline VC levels in our cohort, and therefore, findings may vary in cohorts with a wider range of VC concentrations or with lower overall VC status. Despite these limitations, this study offers valuable insights into the impact of VC supplementation on plasma proteins in T2D.

This study provides the first comprehensive proteomic evidence using label-free LC-MS/MS methods showing that VC supplementation in adults with T2D modulates a diverse range of proteins, offering novel insights into its possible mechanisms of action. Our findings suggest that VC might exert beneficial effects on glycaemic outcomes by attenuating acute-phase response proteins such as SAA1. The downregulation of molecular functions of protein folding chaperones and ATP binding through decreases in heat shock proteins in plasma suggests that VC supplementation mitigates cellular oxidative stress/damage, which might impact clinically on outcomes such as blood pressure. Downregulation of AMY1 also suggests a previously unreported pathway by which VC may influence carbohydrate digestion in T2D. Novel findings, such as the depletion of numerous proteins and pathways not previously reported to be altered in the plasma with VC supplementation, require further research to better understand their biological significance. Our findings are largely hypothesis-generating and targeted research is warranted to clarify the complex interplay of these proteins and to validate their potential as biomarkers and therapeutic targets for the management of T2D and its complications.

## Supplementary information

Supplementary Tables and Figures

## Data Availability

The mass spectrometry proteomics data have been deposited to the ProteomeXchange Consortium via the PRIDE [64] partner repository with the dataset identifier PXD068613.
