# Supplementary material for: Plasma proteomics analysis of vitamin C supplementation in people with type 2 diabetes
Source: Nutr Diabetes. 2026 Jun 27;16:37. doi: 10.1038/s41387-026-00447-5 (PMC13574769; doi:10.1038/s41387-026-00447-5)
Supplement: Supplementary file 1 [file 41387_2026_447_MOESM1_ESM.docx]

**Supplementary Table S1**. All significant protein log2-fold changes and FDR-adjusted p values for main analysis.

| **Gene Symbol** | **Log2 Fold Change** | **P-Value** | **FDR** |
| --- | --- | --- | --- |
| *KDM5B** | *-4.16648* | *3.47E-05* | *4.08E-04* |
| **MYH1** | **-1.51612** | **4.46E-05** | **5.05E-04** |
| **AMY1B;AMY1C;AMY1A** | **-1.3849** | **4.56E-04** | **3.78E-03** |
| **TRAP1** | **-1.17179** | **4.73E-05** | **5.25E-04** |
| **SAA1** | **-1.01131** | **2.16E-04** | **1.96E-03** |
| **DCD** | **-0.98698** | **3.10E-03** | **1.96E-02** |
| **CRP** | **-0.7982** | **1.69E-03** | **1.19E-02** |
| **EIF2B2** | **-0.78986** | **9.96E-07** | **1.97E-05** |
| **HSP90AB1** | **-0.7072** | **1.62E-05** | **2.09E-04** |
| **H4C16** | **-0.69812** | **1.13E-04** | **1.10E-03** |
| **ATP6V1B2** | **-0.60412** | **1.88E-11** | **2.62E-09** |
| *POSTN** | *-0.59759* | 3.33E-03 | 2.08E-02 |
| **ACTA1** | **-0.59614** | **5.73E-03** | **3.23E-02** |
| **HSPA1A;HSPA1B** | **-0.50262** | **3.52E-07** | **7.81E-06** |
| JUP | -0.46164 | 1.14E-03 | 8.52E-03 |
| CPAMD8 | -0.4377 | 3.93E-09 | 1.98E-07 |
| S100A9 | -0.4365 | 3.10E-04 | 2.73E-03 |
| GP5 | -0.40559 | 1.82E-11 | 2.62E-09 |
| AGPAT3 | -0.34581 | 8.62E-06 | 1.29E-04 |
| P4HB | -0.33943 | 3.61E-03 | 2.19E-02 |
| MUS81 | -0.27143 | 3.68E-08 | 9.73E-07 |
| GTF2E1 | -0.26767 | 1.09E-08 | 4.32E-07 |
| B2M | -0.22824 | 3.24E-08 | 8.99E-07 |
| DNAAF1 | -0.21314 | 1.23E-08 | 4.54E-07 |
| IL17D | -0.20213 | 1.85E-15 | 1.03E-12 |
| HABP2 | -0.17994 | 3.65E-03 | 2.19E-02 |
| TTC14 | -0.16772 | 7.17E-04 | 5.74E-03 |
| ATP5F1A | -0.16738 | 8.54E-05 | 8.79E-04 |
| PCSK9 | -0.14739 | 3.68E-03 | 2.19E-02 |
| APOL1 | -0.14108 | 3.62E-03 | 2.19E-02 |
| LECT2 | -0.13813 | 2.85E-10 | 2.13E-08 |
| RSC1A1 | -0.13728 | 5.65E-05 | 6.03E-04 |
| TRAF2 | -0.13017 | 9.16E-06 | 1.34E-04 |
| COL6A1 | -0.11023 | 2.17E-14 | 6.03E-12 |
| MYH8 | -0.10492 | 3.76E-04 | 3.17E-03 |
| IGLC2;IGLC3 | -0.10154 | 1.36E-03 | 1.01E-02 |
| HBS1L | -0.10021 | 2.79E-05 | 3.44E-04 |
| FLNA | -0.08958 | 3.41E-04 | 2.96E-03 |
| EZR | -0.07498 | 3.07E-10 | 2.13E-08 |
| SLC26A2 | -0.07285 | 3.53E-05 | 4.08E-04 |
| UBB;UBC;RPS27A;UBA52 | -0.06177 | 8.56E-05 | 8.79E-04 |
| ANXA2 | -0.05839 | 7.46E-04 | 5.83E-03 |
| ZCCHC8 | -0.04782 | 5.18E-06 | 8.46E-05 |
| CKM | -0.04734 | 3.77E-04 | 3.17E-03 |
| PRDX1 | -0.04613 | 9.90E-06 | 1.41E-04 |
| LAMB1 | -0.0451 | 3.51E-05 | 4.08E-04 |
| NAGA | -0.04492 | 1.64E-08 | 5.37E-07 |
| ANGPTL6 | -0.03424 | 1.11E-10 | 1.23E-08 |
| ATP2A2 | -0.02758 | 5.10E-05 | 5.55E-04 |
| ACTN1 | -0.02542 | 1.79E-05 | 2.26E-04 |
| MBL2 | -0.01377 | 7.96E-07 | 1.64E-05 |
| ARF6 | -0.01197 | 1.09E-08 | 4.32E-07 |
| TPM1 | -0.00978 | 3.28E-09 | 1.82E-07 |
| ATP5F1B | -0.00799 | 4.70E-03 | 2.72E-02 |
| H1-4 | -0.00349 | 1.90E-03 | 1.30E-02 |
| VPS9D1 | 0.00122 | 1.11E-04 | 1.10E-03 |
| RPAP3 | 0.005319 | 1.59E-06 | 2.93E-05 |
| TFRC | 0.015506 | 3.53E-06 | 6.13E-05 |
| TPI1 | 0.017542 | 5.11E-06 | 8.46E-05 |
| CDH1 | 0.03553 | 9.25E-09 | 4.28E-07 |
| PON3 | 0.043173 | 2.64E-03 | 1.74E-02 |
| GSR | 0.04561 | 2.07E-04 | 1.92E-03 |
| IGKV3D-15 | 0.046745 | 8.90E-04 | 6.86E-03 |
| FAM135A | 0.046788 | 8.26E-06 | 1.27E-04 |
| A2M | 0.05263 | 8.12E-03 | 4.46E-02 |
| IGKV1-5 | 0.064637 | 2.85E-03 | 1.84E-02 |
| DSG2 | 0.065647 | 4.87E-08 | 1.23E-06 |
| CETP | 0.074314 | 2.49E-09 | 1.54E-07 |
| IGKV1-37;IGKV1D-37 | 0.075623 | 1.07E-06 | 2.04E-05 |
| A1BG | 0.078019 | 5.76E-03 | 3.23E-02 |
| ENO3 | 0.079302 | 7.39E-07 | 1.58E-05 |
| BLVRB | 0.084005 | 1.03E-05 | 1.41E-04 |
| TTN | 0.084013 | 1.30E-04 | 1.22E-03 |
| LDHB | 0.087755 | 1.04E-05 | 1.41E-04 |
| ARG1 | 0.088958 | 2.48E-06 | 4.44E-05 |
| ADGRG1 | 0.095237 | 5.53E-06 | 8.76E-05 |
| ADAMTS13 | 0.101576 | 1.30E-04 | 1.22E-03 |
| IGKV1-39;IGKV1D-39 | 0.103585 | 7.24E-04 | 5.74E-03 |
| ANPEP | 0.104181 | 1.68E-03 | 1.19E-02 |
| FUCA1 | 0.115511 | 4.31E-03 | 2.52E-02 |
| CNTN1 | 0.116862 | 1.60E-08 | 5.37E-07 |
| IGHM | 0.117273 | 7.30E-03 | 4.05E-02 |
| F10 | 0.11774 | 3.04E-03 | 1.94E-02 |
| IGKJ1 | 0.128244 | 5.14E-03 | 2.94E-02 |
| TNC | 0.128265 | 2.17E-03 | 1.47E-02 |
| CPN2 | 0.129113 | 2.23E-03 | 1.49E-02 |
| LRRC37A2;LRRC37A;LRRC37A3 | 0.131348 | 2.62E-10 | 2.13E-08 |
| IGF2R | 0.132118 | 3.86E-03 | 2.28E-02 |
| TNK1 | 0.135388 | 8.46E-08 | 1.96E-06 |
| LDHA | 0.143205 | 9.40E-04 | 7.15E-03 |
| VCAM1 | 0.145112 | 3.01E-04 | 2.70E-03 |
| VASN | 0.170366 | 5.41E-04 | 4.42E-03 |
| IL6ST | 0.181696 | 1.71E-03 | 1.19E-02 |
| OIT3 | 0.182781 | 5.26E-08 | 1.27E-06 |
| PGK1 | 0.184184 | 2.67E-03 | 1.75E-02 |
| PTPRJ | 0.206251 | 2.93E-08 | 8.57E-07 |
| IGHV3-9 | 0.255465 | 1.35E-05 | 1.78E-04 |
| MYL6 | 0.277945 | 9.88E-05 | 9.97E-04 |
| **CA2** | **0.594351** | **1.62E-03** | **1.16E-02** |
| **HIVEP1** | **0.739016** | **1.58E-03** | **1.16E-02** |
| **ALCAM** | **0.800885** | **2.54E-08** | **7.84E-07** |

(*Bold* values indicate candidates. **Italicised* proteins were removed as candidates due to likely imputation-driven findings; FDR, false discovery rate;)

**Supplementary Table S2.** Candidate proteins in sensitivity analysis investigating VC supplementation vs. control for Period-1 and Period-2 separately.

| **Tier 1 candidates** | **Period 1 (first intervention period)** | | **Period 2 (second intervention period)** | |
| --- | --- | --- | --- | --- |
| **Gene symbol** | **Log2 fold change** | **FDR** | **Log2 fold change** | **FDR** |
| DCD | -1.270 | **4.596E-03** | *0.192* | 4.117E-01 |
| AMY1 | -1.249 | **3.859E-02** | *0.048* | ***2.818E-27*** |
| TRAP1 | -0.972 | **7.160E-03** | *0.035* | 6.135E-01 |
| MYH1 | -0.814 | **2.612E-02** | -0.728 | **1.941E-03** |
| EIF2B2 | -0.630 | **1.387E-02** | -0.778 | **6.548E-04** |
| H4C16 | -0.598 | **8.800E-03** | -0.125 | **1.743E-05** |
| ACTA1 | -0.459 | **3.545E-02** | -0.042 | 3.140E-01 |
| SAA1 | -0.454 | **1.147E-09** | -1.095 | **4.226E-03** |
| HSP90AB1 | -0.371 | **7.748E-03** | -0.017 | 4.037E-01 |
| ALCAM | 0.853 | **1.203E-03** | 0.687 | 3.864E-01 |
|  |  |  |  |  |
| **Tier 2 candidates** | **Period 1 (first intervention period)** | | **Period 2 (second intervention period)** | |
| **Gene symbol** | **Log2 fold change** | **FDR** | **Log2 fold change** | **FDR** |
| ATP6V1B2 | -0.944 | 5.502E-01 | -0.489 | **6.345E-14** |
| HSPA1 | -0.808 | **1.623E-03** | *0.151* | ***3.367E-14*** |
| CA2 | 0.023 | 5.791E-01 | 0.730 | 1.736E-01 |
| CRP^*^ | *0.234* | 4.214E-01 | -1.815 | **7.029E-05** |
| HIVEP1 | 0.388 | 4.367E-01 | 0.378 | **1.717E-02** |
|  |  |  |  |  |

(**Bold** values indicate statistical significance; *italicized* values represent opposite directions of effect to main analysis; ^*^CRP at baseline differed significantly between VC and placebo in Period 1 [log2 fold change = 1.31, FDR = 1.76 x 10^-7^]; FDR, false discovery rate;).

**Supplementary Table S3.** Candidate proteins in “per-protocol” sensitivity analysis (n=19) investigating VC supplementation vs. Control, with participants removed for either medication changes or supplement non-compliance.

| **Tier 1 candidates** |  | |
| --- | --- | --- |
| **Gene symbol** | **Log2 fold change** | **FDR** |
| MYH1 | -1.882 | **4.917E-03** |
| SAA1 | -1.404 | **4.514E-03** |
| TRAP1 | -1.403 | **4.721E-03** |
| AMY1 | -1.383 | **1.360E-03** |
| HSP90AB1 | -1.155 | **1.065E-02** |
| EIF2B2 | -1.116 | **3.688E-04** |
| DCD | -0.993 | 1.356E-01 |
| H4C16 | -0.908 | **2.060E-03** |
| ACTA1 | -0.898 | **4.724E-02** |
| ALCAM | 0.835 | **2.449E-04** |
|  |  |  |
| **Tier 2 candidates** |  | |
| **Gene symbol** | **Log2 fold change** | **FDR** |
| CRP | -1.140 | **2.648E-03** |
| ATP6V1B2^*^ | -1.006 | 2.509E-01 |
| HSPA1 | -0.764 | **1.018E-03** |
| HIVEP1^*^ | 0.144 | 7.787E-01 |
| CA2 | 0.198 | 2.760E-01 |
|  |  |  |

(**Bold** values indicate statistically significant; ^*^calculated based on non-filtered/non-imputed data due to their removal after filtering out >40% missing data; FDR – false discovery rate)

**Supplementary Table S4**: Top 5 gene ontology enriched terms for top 20 correlated baseline proteins* with intervention response for targeted outcomes

| **Outcome measure** | |  |  |  |  |
| --- | --- | --- | --- | --- | --- |
| **Total Postprandial Area Under the Curve** | |  |  |  |  |
| **Biological Process (Gene Ontology)** | | | |  |  |
| **GO-term** | **description** | **count in network** | **strength** | **signal** | **FDR** |
| GO:0098883 | Synapse pruning | 3 of 11 | 2.69 | 1.8 | 0.00051 |
| GO:0006956 | Complement activation | 4 of 60 | 2.08 | 1.69 | 0.00051 |
| GO:0006959 | Humoral immune response | 5 of 268 | 1.52 | 1.32 | 0.00083 |
| GO:0006958 | Complement activation, classical pathway | 3 of 40 | 2.13 | 1.22 | 0.0049 |
| GO:0045087 | Innate immune response | 5 of 754 | 1.07 | 0.53 | 0.0499 |
|  |  |  |  |  |  |
| **Cellular Component (Gene Ontology)** | | | |  |  |
| **GO-term** | **description** | **count in network** | **strength** | **signal** | **FDR** |
| GO:0062167 | Complement component C1q complex | 3 of 3 | 3.25 | 3.07 | 2.81E-06 |
| GO:0005602 | Complement component C1 complex | 3 of 5 | 3.03 | 2.98 | 3.70E-06 |
| GO:0005581 | Collagen trimer | 5 of 95 | 1.97 | 2.6 | 2.81E-06 |
| GO:0062023 | Collagen-containing extracellular matrix | 6 of 407 | 1.42 | 1.69 | 1.41E-05 |
| GO:0072562 | Blood microparticle | 3 of 118 | 1.66 | 1.01 | 0.0082 |
| **Time in Hyperglycaemia** | | |  |  |  |
| **Biological Process (Gene Ontology)** | | | |  |  |
| **GO-term** | **description** | **count in network** | **strength** | **signal** | **FDR** |
| GO:0006958 | Complement activation, classical pathway | 6 of 40 | 2.32 | 3.84 | 5.34E-09 |
| GO:0045087 | Innate immune response | 8 of 754 | 1.17 | 1.28 | 1.84E-05 |
| GO:0006952 | Defense response | 10 of 1394 | 1 | 1.09 | 4.90E-06 |
| GO:0098883 | Synapse pruning | 2 of 11 | 2.41 | 0.86 | 0.0204 |
| GO:0002682 | Regulation of immune system process | 8 of 1438 | 0.89 | 0.73 | 0.0012 |
|  |  |  |  |  |  |
| **Cellular Component (Gene Ontology)** | | | |  |  |
| **GO-term** | **description** | **count in network** | **strength** | **signal** | **FDR** |
| GO:0072562 | Blood microparticle | 8 of 118 | 1.98 | 4.24 | 1.32E-11 |
| GO:0005602 | Complement component C1 complex | 4 of 5 | 3.05 | 3.9 | 2.05E-08 |
| GO:0062167 | Complement component C1q complex | 2 of 3 | 2.97 | 1.4 | 0.0019 |
| GO:0005615 | Extracellular space | 13 of 3247 | 0.75 | 0.77 | 5.49E-07 |
| **Average 24h glucose** | |  |  |  |  |
| **Biological Process (Gene Ontology)** | | | |  |  |
| **GO-term** | **description** | **count in network** | **strength** | **signal** | **FDR** |
| GO:0006958 | Complement activation, classical pathway | 4 of 40 | 2.12 | 1.58 | 0.00045 |
| GO:0098883 | Synapse pruning | 3 of 11 | 2.55 | 1.57 | 0.00067 |
| GO:0051346 | Negative regulation of hydrolase activity | 6 of 354 | 1.35 | 1.15 | 0.00067 |
| GO:0006959 | Humoral immune response | 5 of 268 | 1.39 | 1.08 | 0.0015 |
| GO:0030162 | Regulation of proteolysis | 7 of 739 | 1.09 | 0.91 | 0.001 |
|  |  |  |  |  |  |
| **Cellular Component (Gene Ontology)** | | | |  |  |
| **GO-term** | **description** | **count in network** | **strength** | **signal** | **FDR** |
| GO:0005602 | Complement component C1 complex | 4 of 5 | 3.02 | 3.83 | 1.86E-08 |
| GO:0072562 | Blood microparticle | 7 of 118 | 1.89 | 3.28 | 3.80E-09 |
| GO:0062167 | Complement component C1q complex | 3 of 3 | 3.12 | 2.77 | 2.91E-06 |
| GO:0005581 | Collagen trimer | 4 of 95 | 1.74 | 1.62 | 0.00015 |
| GO:0062023 | Collagen-containing extracellular matrix | 6 of 407 | 1.29 | 1.27 | 0.00012 |
| **Systolic blood pressure** | |  |  |  |  |
| **Biological Process (Gene Ontology)** | | | |  |  |
| **GO-term** | **description** | **count in network** | **strength** | **signal** | **FDR** |
| GO:0006958 | Complement activation, classical pathway | 6 of 40 | 2.17 | 3.07 | 4.05E-08 |
| GO:0002449 | Lymphocyte mediated immunity | 8 of 159 | 1.7 | 2.54 | 4.05E-08 |
| GO:0006957 | Complement activation, alternative pathway | 4 of 16 | 2.39 | 2.31 | 5.79E-06 |
| GO:0002460 | Adaptive immune response based on somatic recombination of immune receptors built from immunoglobulin superfamily domains | 7 of 169 | 1.61 | 2.12 | 6.30E-07 |
| GO:0019835 | Cytolysis | 4 of 23 | 2.23 | 2.08 | 1.69E-05 |
|  |  |  |  |  |  |
| **Cellular Component (Gene Ontology)** | | | |  |  |
| **GO-term** | **description** | **count in network** | **strength** | **signal** | **FDR** |
| GO:0005579 | Membrane attack complex | 4 of 7 | 2.75 | 3.24 | 6.47E-08 |
| GO:0072562 | Blood microparticle | 5 of 118 | 1.62 | 1.71 | 2.33E-05 |
| GO:0005788 | Endoplasmic reticulum lumen | 7 of 312 | 1.34 | 1.59 | 4.00E-06 |
| GO:1903561 | Extracellular vesicle | 20 of 2120 | 0.97 | 1.48 | 9.71E-17 |
| GO:0070062 | Extracellular exosome | 19 of 2096 | 0.95 | 1.4 | 3.24E-15 |
| **Diastolic blood pressure** | |  |  |  |  |
| **Biological Process (Gene Ontology)** | | | |  |  |
| **GO-term** | **description** | **count in network** | **strength** | **signal** | **FDR** |
| GO:0006957 | Complement activation, alternative pathway | 3 of 16 | 2.31 | 1.41 | 0.00087 |
| GO:0006958 | Complement activation, classical pathway | 4 of 40 | 2.04 | 1.38 | 0.00083 |
| GO:0019835 | Cytolysis | 3 of 23 | 2.15 | 1.25 | 0.0018 |
| GO:0006959 | Humoral immune response | 6 of 268 | 1.39 | 1.12 | 0.00083 |
| GO:0045087 | Innate immune response | 8 of 754 | 1.06 | 0.89 | 0.00083 |
|  |  |  |  |  |  |
| **Cellular Component (Gene Ontology)** | | | |  |  |
| **GO-term** | **description** | **count in network** | **strength** | **signal** | **FDR** |
| GO:0005579 | Membrane attack complex | 3 of 7 | 2.67 | 2.17 | 2.23E-05 |
| GO:0072562 | Blood microparticle | 4 of 118 | 1.57 | 1.22 | 0.00082 |
| GO:0070062 | Extracellular exosome | 13 of 2096 | 0.83 | 0.9 | 8.07E-07 |
| GO:0005615 | Extracellular space | 16 of 3247 | 0.73 | 0.8 | 6.86E-08 |
| GO:0005576 | Extracellular region | 17 of 4175 | 0.65 | 0.69 | 6.86E-08 |

(*top 20 proteins are those with the lowest p values determined via Spearman correlation, and with no missing data; *strength* describes how large the enrichment effect is; *signal* is defined as a weighted harmonic mean between the observed/expected; FDR, false discovery rate;).

**Figure S1**. Protein group data completeness across samples showing missing data values (A) and number of protein groups found across all samples noting complete, 50% shared, sparse, and unique identifications (B) (Figure generated using Spectronaut v19.0).

**Figure S2**. Original (A) and normalised (B) peptide signal responses across samples (Adapted figures generated using Spectronaut v19.0).

**Figure S3**. % Coefficient of Variation for each condition. Data shows median, mean, interquartile range, and range of data within each condition (Figure generated using Spectronaut v19.0).

**Figure S4**. Principal Component Analysis including comparison of all conditions (Figure generated using Spectronaut v19.0).

**Figure S5**. Correlations of aggregated protein abundance at baseline for enriched Gene Ontology term Complement activation (classical pathway; n=30 proteins) and clinical outcomes responsive to VC supplementation. Δ values represent treatment response (Δ VC supplementation minus Δ placebo supplementation); PPG AUC – postprandial glucose area under the curve; SBP – systolic blood pressure; DBP – diastolic blood pressure.

 **Figure S6.** Correlation of aggregate protein abundance at baseline for GO term C1 complex abundance (n=5 proteins) and glycaemic-related clinical outcomes (A-C) and correlation of aggregate protein abundance at baseline for GO term Membrane attack complex (n=7 proteins) and blood pressure-related outcomes (D-E Δ values represent treatment response (Δ VC supplementation minus Δ placebo supplementation); PPG AUC – postprandial glucose area under the curve; SBP – systolic blood pressure; DBP – diastolic blood pressure.
